# Including the Public in Public eHealth: The Need for Community Participation in the Development of State-Sponsored COVID-19–Related Mobile Apps

**DOI:** 10.2196/30872

**Published:** 2022-03-09

**Authors:** Muhammed Yassin Idris, Maya Korin, Faven Araya, Sayeeda Chowdhury, Patty Medina, Larissa Cruz, Trey-Rashad Hawkins, Humberto Brown, Luz Claudio

**Affiliations:** 1 Department of Environmental Medicine and Public Health Icahn School of Medicine at Mount Sinai New York, NY United States; 2 Department of Medicine Morehouse School of Medicine Atlanta, GA United States; 3 Arthur Ashe Institute for Urban Health New York, NY United States

**Keywords:** mobile apps, COVID-19, CBPR, digital health, eHealth, community health, health disparities

## Abstract

The COVID-19 pandemic has overwhelmed health care systems worldwide, particularly in underresourced communities of color with a high prevalence of pre-existing health conditions. Many state governments and health care entities responded by increasing their capacity for telemedicine and disease tracking and creating mobile apps for dissemination of medical information. Our experiences with state-sponsored apps suggest that because many of these eHealth tools did not include community participation, they inadvertently contributed to widening digital health disparities. We propose that, as eHealth tools continue to expand as a form of health care, more attention needs to be given to their equitable distribution, accessibility, and usage. In this viewpoint collaboratively written by a minority-serving community-based organization and an eHealth academic research team, we present our experience participating in a community advisory board working on the dissemination of the COVID Alert NY mobile app to illustrate the importance of public participation in app development. We also provide practical recommendations on how to involve community representatives in the app development process. We propose that transparency and community involvement in the process of app development ultimately increases buy-in, trust, and usage of digital technology in communities where they are needed most.

## Introduction

Since the start of the COVID-19 outbreak, eHealth tools have been rapidly deployed, including telemedicine, mobile health apps, and wearable technologies [1,2]. These eHealth tools can be used to reduce and monitor disease. However, they may inadvertently result in increasing underlying inequalities by unevenly benefiting individuals who are better able to access new information, adopt technologies early on, and have more resources to pay for these new innovations [3]. As Crawford and Serhal (2020) [2] highlight, “digital health technologies interact with social, cultural, and economic realities and with social determinants of health to indirectly contribute to health inequity.” Barriers to using eHealth technologies in underserved communities also include a lack of perceived value, limited digital and health literacy, and a lack of relevance [4,5].

Community-based partnerships are key to addressing these social determinants that serve as barriers to closing the digital divide and using technology to promote health equity [6]. In this viewpoint, when we refer to community, we are distinguishing between a “user” community defined as the client or consumer of a particular technology, platform, or service and the communities (of color) as defined by the collective sector of the public that is disproportionately impacted by health disparities and that stands to benefit most by use of this technology. The latter is characterized by a shared sense of identity, understanding, or geographical distribution, but may or may not self-select into a user community [7]. As with the adoption of any new product or innovation, involvement of communities throughout the eHealth development process influences awareness of need in underserved communities as well as decisions around initial use, adoption, rejection, and continued use of eHealth innovations [8].

The goal of this viewpoint is to illustrate the importance of community involvement throughout the development of state-sponsored eHealth apps. We have chosen to focus on state-funded eHealth apps because these are funded by taxpayer dollars and should be responsive to public health needs. In what follows, we first present preliminary data on the proliferation of state-sponsored COVID-19–related contact tracing apps between February 26, 2020, and December 31, 2020. We then speak to our experience on the New York City (NYC) Health + Hospitals’ community advisory board (CAB) during the rollout of the COVID Alert NY mobile app. Our experience shows how community involvement has practical implications on trust and buy-in from community-based organizations and, by extension, from communities disproportionately impacted by health inequities. We also provide some practical recommendations for developers on when and how to involve community representatives in their development process.

## The Proliferation of State-Sponsored COVID-19 Contact Tracing Mobile Apps

**Figure 1:**
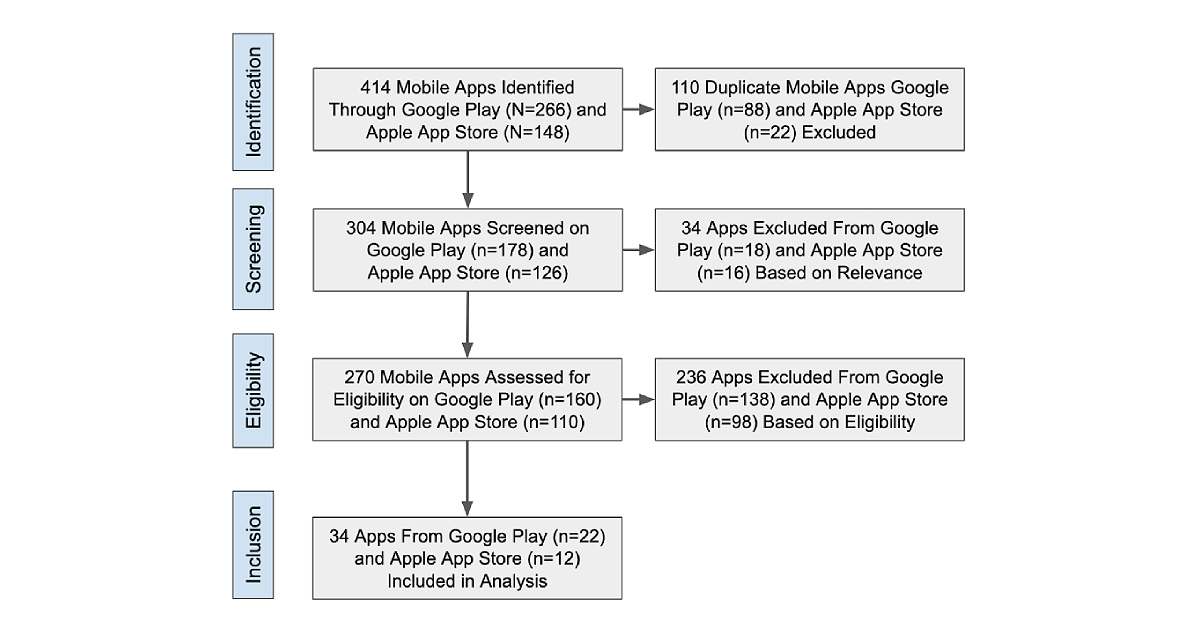
Selection process of COVID-19 apps from Apple App Store and Google Play Store.

We conducted a systematic search of state-sponsored COVID-19 apps on the Apple App Store and Google Play Store. We examined all COVID-19–related health apps released between February 26, 2020, and December 31, 2020. Following the methods outlined by Davalbhakta et al [9], we relied on the following keywords in the Apple App Store and Google Play Store: “Covid,” “Corona,” “Pandemic,” “Covid-19,” “SARSCOV2,” “coronavirus,” “2019-nCoV,” “Covid-19 tracker,” “Stop COVID,” and “c-19.” Two reviewers (TRH and SC) screened apps for relevance and eligibility for inclusion, including whether the apps were state-sponsored. Where there was ambiguity or disagreement around relevance and eligibility, review of the app was escalated to the lead investigator (MYI) for adjudication. Figure 1 provides a flowchart outlining our search and selection process.

Following the 3 most common categories in schemas used in previous publications of COVID-19 apps [10-12], we categorized the apps in our search based on 3 distinct functionalities: (1) contact tracing/exposure notification, (2) symptom checking, and (3) information dissemination. Contact tracing and exposure notification functionality allows users to turn on exposure notifications and be alerted when users encounter (anonymous) others who tested positive in their location. Symptom checking allows users to enter symptoms along with some simple answers to questions and reveals options for next steps regarding the likelihood of COVID-19 infection. Information dissemination functionality in eHealth apps focuses on providing facts about COVID-19, good hygiene practices, and guidelines to follow, like social distancing and the importance of wearing face masks, how to access resources, and other types of relevant information.

**Figure 2:**
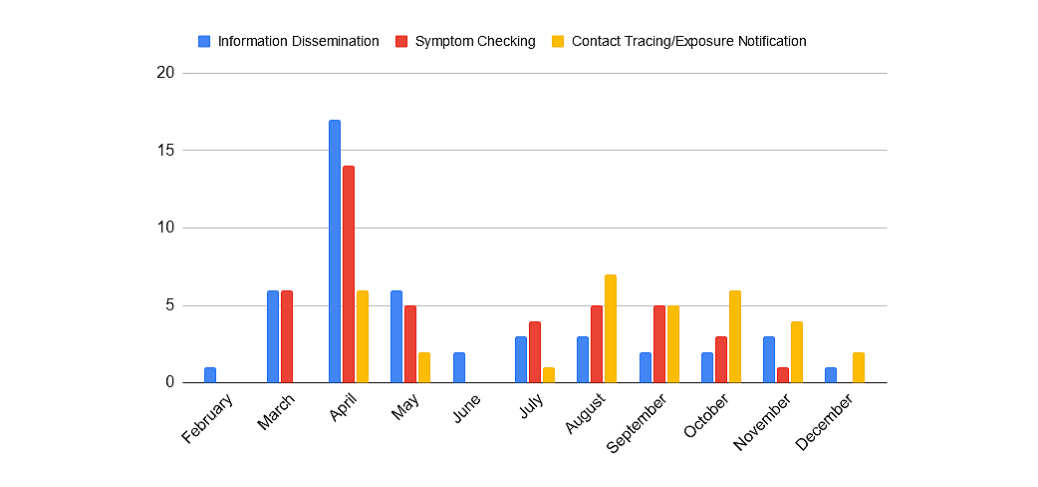
Count of apps released over time by category.

A total of 34 apps (12 Apple, 22 Google) met the inclusion criteria. Of them, 12% (n=4) only provided information to users (eg, suggested resources, provided updates, and delivered public service announcements through push notifications), 3% (n=1) only collected data from users (eg, symptom tracker that will determine whether a person may need further assessment or testing for COVID-19), and 85% (n=29) of apps provided both information and collected data. In addition, 41% (n=14) of all apps reviewed included at least one feature for checking COVID-19–related symptoms, 77% (n=26) included functionality around contact tracing and exposure notification, and 50% (n=17) provided some information dissemination functionality. Table 1 shows the apps by category (eg, checking symptoms, contact tracing/exposure notification, and information dissemination) and Figure 2 provides a visualization of the number of COVID-19 apps released by category between February 2020 and December 2020.

**Table 1:** List of state-sponsored COVID-19 apps by category.

Name of app	Approximate date released	Operating system	Contact tracing and exposure notification	Symptom checking	Information dissemination
ABTrace Together	4/1/2020	iOS	✓		
AlohaSafe Alert	11/10/2020	iOS	✓		✓
ArriveCAN	4/21/2020	iOS		✓	
BC COVID-19 Support	5/21/2020	iOS		✓	✓
CA Notify	12/11/2020	Android	✓		
Canada COVID-19 (COVID Alert)	7/30/2020	iOS		✓	✓
Care19 Alert	8/10/2020	Both	✓		
Care19 Diary	4/19/2020	Android	✓	✓	✓
CO Exposure Notification	10/23/2020	Android	✓		
CombatCOVID MDC	8/20/2020	Both	✓		✓
COVID Alert	7/28/2020	Both	✓		
Covid Alert CT	10/30/2020	Android	✓		
Covid Alert DE	9/8/2020	Both	✓	✓	
Covid Alert NJ	9/30/2020	Both	✓	✓	✓
Covid Alert NY	9/30/2020	Both	✓	✓	
Covid Alert PA	9/11/2020	Both	✓	✓	✓
Covid Trace Nevada	8/17/2020	Both	✓	✓	✓
Covid View	12/29/2020	iOS			✓
Covid Watch Arizona	08/17/2020	Both	✓		
COVID-19 Virginia Resources	4/27/2020	Both			✓
Covid-19 Wisconsin Connect	5/14/2020	iOS			✓
Covidaware MN	11/16/2020	Both	✓		✓
CovidWise	8/7/2020	Both	✓		✓
Crush Covid RI	5/15/2020	Both	✓	✓	✓
DC Can	10/23/2020	Android	✓		
GuideSafe	8/11/2020	Both	✓	✓	
Healthy Together	4/1/2020	Both	✓	✓	✓
MD Covid Alert	11/5/2020	Android	✓		
MI Covid Alert	10/1/2020	Both	✓	✓	
NJ COVID 19	3/31/2020	iOS			✓
SlowCovidNC	9/16/2020	Both	✓		
Stronger than C19	4/25/2020	Both		✓	✓
WA Notify	11/25/2020	Android	✓		
WI Exposure Notification	12/20/2020	Android	✓		

A clear trend emerges where most apps released early in the pandemic were primarily focused on symptom checking and information dissemination. Starting in August 2020, however, we see fewer apps being released overall, but more activity around contact tracing apps. This trend corresponds with the release of many apps by local and national governments using Bluetooth-based exposure notification incorporating a system codeveloped by Google and Apple [13]. The technology allows users to turn on exposure notifications and be alerted when users encounter (anonymous) others who tested positive in their location. This collaboration between high-tech and public health organizations has been hailed as an exemplar of technology partnership for social good [14], yet little is publicly known about who is using these state-sponsored contact tracing apps.

## COVID Alert NY Mobile App as a Case In Point

In NYC, there were 3 entities that played a significant role in the development and dissemination of the COVID Alert NY mobile app: the New York State Department of Health, NYC Department of Health and Mental Hygiene (DOHMH), and NYC Health + Hospitals, the largest municipal health care system in the United States, serving almost 500,000 uninsured NYC residents. Despite caution by community-based organizations and advocates early in the pandemic, there was not enough data to document health disparities related to COVID-19 morbidity and mortality [15]. It was clear on the ground that COVID-19 testing sites were not accessible to NYC communities of color [16]. Hesitancy around testing was further amplified by inadequate care where patients of color would, despite showing symptoms, be told that they were fine and be sent home on multiple occasions.

Once the data were collected and racial and ethnic disparities became apparent, an NYC Test and Trace CAB was organized to provide input on COVID-19–related efforts in NYC in May 2020. The CAB, which meets weekly with city health officials, was organized under the auspices of NYC Health + Hospitals and consisted of 71 members representing a broad range of organizations across all 5 boroughs [17]. The CAB was instrumental in directing where testing sites should be deployed and providing guidance on effective strategies for communicating critical information to community members while addressing language barriers and concerns around health literacy. The CAB also played a vital role in sharing lived experiences of community members to inform and reinforce community-based recommendations.

Although the DOHMH and NYC Health + Hospitals were responsive to the CAB’s questions, concerns, and counsel around testing, there was no CAB input into the design, development, and dissemination of the COVID Alert NY mobile app for contact tracing and exposure notification, which was presented to the CAB in September 2020. Given the government’s responsibility for public health, state and local authorities must be responsive to the best interest of their constituents and the public. Recognizing this responsibility, it is therefore essential to establish and implement community-driven processes that incorporate and prioritize the needs and concerns of disproportionately impacted and underserved communities. Our experience at the grassroots suggests that when there is proper community consultation, what follows is more engagement with, usage of, and penetration of government-led interventions.

As the epicenter of the pandemic in the United States in early 2020, NYC’s response to the COVID-19 pandemic was positioned to model community-engaged practices that combat health disparities and promote health equity. Community-based organizations and advocates on the CAB expressed concerns around the digital divide and trust in state-sponsored apps within communities of color early in the pandemic. It is important to note here that the release of the COVID Alert NY mobile app was coming on the heel of Public Charge, in which public officials could deny applications for lawful immigration if they determined the applicant has used or will depend on public benefits [18]. The app was developed by the Department of Health and it was shared with the CAB to disseminate by DOHMH and NYC Health + Hospitals. Despite expressing concerns around privacy and confidentiality, the CAB was given an app that was developed without community input and was being asked to disseminate it without having the ability to incorporate community feedback. As a result of this lack of community involvement, the CAB was reluctant to endorse or share the COVID Alert NY mobile app within our communities.

## A Community-Based Participatory Research Approach to Health App Development in Underserved Communities

Developers of COVID-19 mobile apps must address impediments to eHealth tool utilization among underserved and disproportionately impacted communities, including access, privacy, and confidentiality, poor eHealth literacy, and language barriers [2,19]. There are numerous frameworks for incorporating community input into the research and development of programs to tackle COVID-19 health disparities. One relevant framework is participatory app design, which involves affected stakeholders from the inception of the project in designing solutions that identify and incorporate the community’s unique needs into the app development [20]. Similarly, user-centered designs provide a framework to better understand who users are, as well as their goals, experiences, and expectations, to ensure users are kept at the center of the design process [21].

Our work focuses on using a community-based participatory research (CBPR) lens to involve community representatives in eHealth app development. CBPR is a collaborative approach that emphasizes long-term partnerships between communities and academics to ensure equity in each aspect of the research and development process [22,23]. For mobile apps to be able to address health disparities, development should include incorporating communities in the problem definition and design, technical and content development, deployment, evaluation, and dissemination of results to stakeholders.

Unlike participatory and user-centered design, a CBPR approach extends beyond incorporating a community perspective into technology design and aims to ensure equity throughout the entire process of design, development, and deployment [24-28]. These principles include understanding communities' resources and technical capacity, defining interactive processes that are responsive to community needs, equitable decision-making, and building collaboration in the design, development, deployment, and transparency around technology-related outputs, ownership, and maintenance [28]. We expand the principle around technology-related outputs to also include transparency around data collection and use. The goal of CBPR is not only to build something useful, but also to improve public health through an iterative and sustainable process where communities of color are kept front and center.

## Practical Recommendations on When and How to Get Community Involved

Our experience serving on the NYC Test and Trace CAB illustrates how transparency and community involvement in the creation of COVID-19–related apps have practical implications for buy-in from community-based organizations and, by extension, from communities disproportionately impacted by COVID-19. Community-based organizations that have the public’s trust due to years of work at the grassroots level, especially those representing and serving communities of color, rely on information about the extent to which the communities they serve were involved in the development of eHealth tools to assess whether to promote these solutions within the communities they serve. We recommend that developers work closely with community-based organizations who can serve as trusted public brokers and can help facilitate community involvement in all phases of app development. In the design phase of the development process, we recommend that app developers work alongside community-based organizations to (1) complete a need-based assessment before/while designing the app, (2) solicit regular community feedback on low- and high-fidelity prototypes, and (3) clearly identify and attribute where community feedback was incorporated. During the development phase, we recommend that developers (4) involve community members in the technical development/testing of the app, (5) involve community members in the development/review of content, and (6) conduct a focus group of community members and leaders to demo a prototype and discuss deployment of the app. Finally, for deployment, we recommend that developers (7) cocreate an evaluation plan (with a corresponding logic model) with a community-based organization partner before the app is deployed and (8) complete the evaluation with the involvement of the community-based organization partner and present results publicly (ie, through publications, presentations) once an app is deployed. We also recommend (9) incentivizing involvement by compensating community members for the opportunity cost of participating in needs-based assessments, focus groups, and program evaluation.

## Discussion and Conclusions

Although other publications have reviewed COVID-19–related mobile apps, no reviews have considered community involvement in their design, development, and dissemination. Ming et al [29] provided an overview of features and functionality of 223 mobile health apps released in the early days of the pandemic on public app stores. Salehinejad et al [30] used the Mobile App Rating Scale to assess the acceptability of quality, content, and functionality of COVID-19–related apps found on the Google Play Store and Apple App Store. In addition, 2 other studies reviewed the literature to identify COVID-19 apps and reviewed apps with features ranging from information dissemination and risk/symptom assessment to contact tracing [10,31]. In line with the studies outlined above, we found that the majority of COVID-19 apps focused on symptom tracking, followed by information dissemination, and then contact tracing and exposure notification. Almalki and Giannicchi [32] provided an overview and taxonomy of COVID-19 apps through September 2020 and found that the majority of apps they reviewed were contact tracing and exposure notification apps developed by governments or national authorities. Unlike previous studies, we focused on state-sponsored apps and provided the first comprehensive search of apps through December 2021.

In this viewpoint, we argue that COVID-19–related eHealth tools funded by taxpayer dollars should involve community-based organizations and advocates in the design, development, and dissemination given the government’s responsibility for the entire public’s health. Community-based organizations assess whether to promote eHealth tools to the communities they serve. In this way, our experiences suggest that transparency and community involvement in the process of app development increase buy-in, trust, and usage. As our personal experience illustrates, when affected communities are not included, this can lead to a lack of buy-in and trust, as well as a lack of community participation in the diffusion of eHealth innovations among constituents of those communities [21].

López et al [31] argue that to “harness its true potential and make the greatest difference, [health information technologies] need to be (1) designed with components that focus on the identification and eliminations of disparities...and (2) tailored to the needs of diverse populations.” The authors point to user-centered design as a framework for incorporating communities of color into the design, development, and deployment of mobile health apps to achieve more equitable and better health outcomes. However, we encourage researchers and practitioners to use a CBPR lens to include communities of color, who are often disproportionately impacted by the COVID-19 pandemic, because this approach goes beyond involving individual “users.” Rather, this CBPR approach includes the broader community, with the goal of improving public health and enhancing community capacity by supporting participation and establishing sustainable programs.

A lack of input from communities of color at every step of the eHealth tool development process contributes to bias. That is, small choices made throughout the design, development, and dissemination process have a large collective impact on the perceptions of adopters in underserved communities of the relative advantage, compatibility with values and experiences, and complexity of use, and the extent to which eHealth tools can be tested or provide tangible benefits [33]. When technology is not designed around the needs, expectations, values, and experiences of individual users, this can lead to a lack of adoption. Within the context of eHealth, this lack of inclusive design results in a lack of diffusion of these innovations within underserved communities, which ultimately exacerbates health disparities [34]. If we truly recognize that health disparities exist, we must ensure that underserved communities feel comfortable with eHealth apps to realize their full potential.

## Abbreviations

CAB: community advisory board
CBPR: community-based participatory research
DOHMH: Department of Health and Mental Hygiene
NYC: New York City
